# Korea HIV/AIDS Cohort Study: study design and baseline characteristics

**DOI:** 10.4178/epih.e2018023

**Published:** 2018-06-06

**Authors:** Bo Youl Choi, Jun Yong Choi, Sang Hoon Han, Sang Il Kim, Mee-Kyung Kee, Min Ja Kim, Shin-Woo Kim, Sung Soon Kim, Yu-Mi Kim, Nam Su Ku, Jin-Soo Lee, Joo-Shil Lee, Yunsu Choi, Kyong Sil Park, Joon Young Song, Jun Hee Woo, Moon Won Kang, June Kim

**Affiliations:** 1 Department of Preventive Medicine, Hanyang university College of Medicine, Seoul, Korea; 2 Department of Internal Medicine and AIDS Research Institute, Yonsei University College of Medicine, Seoul, Korea; 3 Division of Infectious Disease, Department of Internal Medicine, Seoul St. Mary’s Hospital, College of Medicine, The Catholic University of Korea, Seoul, Korea; 4 Division of Viral Disease Research, Center for Infectious Diseases Research, Korea, National Institute of Health, Cheongju, Korea; 5 Division of Infectious Diseases, Department of Internal Medicine, Korea University College of Medicine, Seoul, Korea; 6 Department of Internal Medicine, Kyungpook National University School of Medicine, Daegu, Korea; 7 Center for Infectious Diseases Research, Korea National Institute of Health, Cheongju, Korea; 8 Department of Preventive Medicine, Dong-A University College of Medicine, Busan, Korea; 9 Department of Internal Medicine, Inha University College of Medicine, Incheon, Korea; 10 Center for Immunology and Pathology, Cheongju, Korea; 11 Department of Nursing, Hanyang University School of Nursing, Seoul, Korea; 12 Department of Internal Medicine, Korea University College of Medicine, Seoul, Korea; 13 Department of Infectious Diseases, University of Ulsan College of Medicine, Seoul, Korea

**Keywords:** HIV/AIDS, Cohort studies, Sexually transmitted diseases, Communicable diseases

## Abstract

The number of persons infected by HIV/AIDS has consistently increased in Korea since the first case of HIV/AIDS infection in 1985 and reached 15,208 by 2016. About 1,100 new patients with HIV/ AIDS infections have emerged every year since 2013. In Korea, the Korea HIV/AIDS Cohort Study was established for the evidenced-based prevention, treatment, and effective management of patients infected with human immunodeficiency virus (HIV) in December 2006. This study monitored 1,438 patients, who accounted for about 10% of all patients with HIV/AIDS in Korea, for 10 years with the following aims: (1) to develop an administrative system for the establishment of a HIV/AIDS cohort-based study; (2) to standardize methodologies and the case report forms; and (3) to standardize multi-cohort data and develop a data cleaning method. This study aims to monitor at least 1,000 patients (excluding those for whom investigation had been completed) per year (estimated number of patients who can be monitored by January 2018: 939). By December 2016, the sex distribution was 93.3% for men, and 6.7% for women (gender ratio, 13.9:1.0), and 98.9% of all participants were Korean. More than 50.0% of the participants were confirmed as HIV positive after 2006. This study reports competitive, long-term research that aimed to develop policies for the prevention of chronic infectious diseases for patients with HIV. The data collected over the last decade will be used to develop indices for HIV treatment and health promotion.

## INTRODUCTION

Since the recognition of acquired immune deficiency syndrome (AIDS), which gave rise to pneumocystis pneumonia and Kaposi’s sarcoma in young homosexual men across many cities, including Los Angeles, in the US in 1980 to 1981, the pathogenic organism have been isolated from patients with this syndrome and named lymphadenopathy-associated virus in 1983 [1-3]. Researchers named this immunodeficiency disorder as AIDS, which is caused by human immunodeficiency virus (HIV) [4-6].

In the US, the Multicenter AIDS Cohort study was conducted on high-risk heterosexual and homosexual men in 1983, to understand the disease progression from HIV infection to AIDS expression and death [7]. The Amsterdam cohort study of the Netherlands [8], and the Swiss HIV cohort study of Switzerland were subsequently performed in 1984 and 1988, respectively [9]. The results of HIV/AIDS cohort studies from around the world, which were established in the early period after the recognition of AIDS, showed that the route of infection, immunological characteristics, characteristics of opportunistic infections, cause of death, and AIDS pathogenesis vary with country and race [10-12]. In 2003, Brazil established the HIV-Brazil Cohort and has been monitoring patients with HIV across 26 health facilities [13].

According to the recent data released by the Joint United Nations Programme on HIV/AIDS, there were about 36.7 million adult survivors of HIV/AIDS, 1.8 million new persons infected by HIV/ AIDS, and one million deaths associated with AIDS in 2016 [14].

In Korea, since the first report of HIV-positive patients (one Korean, and one foreigner) in 1985, the cumulative number of patients with HIV has increased to 15,208 (13,584 Koreans, and 1,624 foreigners) by 2016, over the last three decades. Of the 13,584 Koreans infected, 12,606 (92.8%) were men and 978 (7.2%) were women; the ratio of HIV-infected men is markedly higher. The cumulative mortality rate is 15.8%, with 2,134 mortalities of the 13,584 patients, 11,439 survivors of HIV/AIDS in 2016.

Although the number of newly infected patients has been decreasing, the number of HIV-infected patients has increased 4 times since 2000 in Korea, and over 1,000 newly infected patients have emerged every year since 2013 [14,15]. Most domestic studies on HIV/AIDS have been conducted on patients from specific hospitals and have focused on assessing treatment effectiveness [16,17].

The goal of this study was to understand the natural progression from AIDS onset until death, in the early period of HIV infection in Korean patients with HIV/AIDS who exhibit different epidemiological characteristics from foreigners. Additionally, to develop a management and treatment guideline for HIV/AIDS by investigating the epidemiological and clinical characteristics of these patients along with the identifying the factors that influence these characteristics. We started with the Korea HIV/AIDS Cohort Study (KoCosHIV), in which 15 medical institutions that have been treating patients with HIV/AIDS across the country since 2006 participated. A cohort of 1,438 patients has been established, and the patients were repeatedly surveyed.

## MATERIALS AND METHODS

### Participating hospitals

A total of 21 hospitals participated in the KoCosHIV, from December 2006 to December 2016. Of these, 15 mid-, and largescale general hospitals currently operate across six cities (2018).

**Figure 1.**
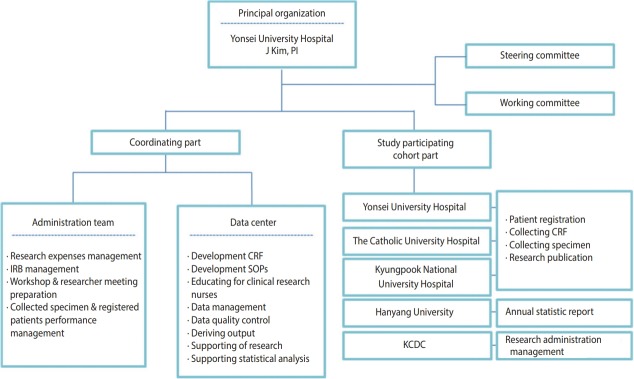
Schematic flow chart of Korea HIV/AIDS Cohort Study organization. PI, principal investigator; IRB, institutional review board; CRF, case report form; SOP, standard operating procedures; KCDC, Korea Centers Disease Control and Prevention.

This study was conducted by a research director, researchers from three research centers, an epidemiological team, and the department of viral diseases of the Korea National Institute of Health. The research director was responsible for the administrative tasks related to the research including conducting research, obtaining institutional review board (IRB) approval, and collecting data. The epidemiology research team was responsible for tasks related to data utilization, such as developing a standardized survey questionnaire and guideline, data cleansing, epidemiological consulting, providing and conducting statistical analyses. The Korea National Institute of Health has the rights to manage the consent form and use data to keep track of the yearly research progress, assign cohort management numbers, conduct participantbased repeated investigation, and manage biological resource samples (Figure 1). The administrative/clinical practice committee regularly met to share opinions related to the research and revised/ improved research tools and indices to make effective progress.

### Subjects

**Figure 2.**
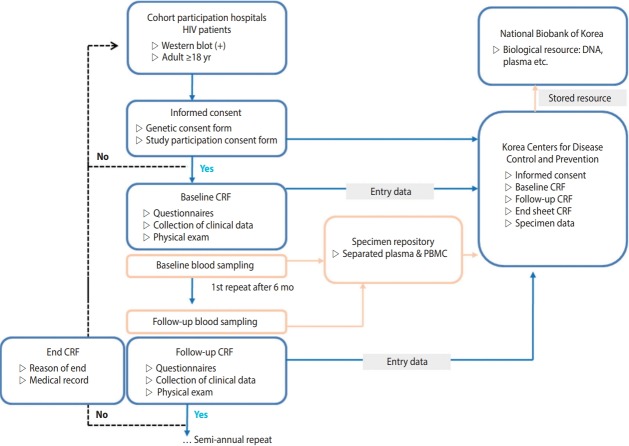
Korea HIV/AIDS Cohort Study design. HIV, human immunodeficiency virus; CRF, case report form; PBMC, peripheral blood mononuclear cell.

HIV-infected Korean adults aged 18 years or older, who were confirmed as HIV positive by HIV Western blot, registered at the Korea Centers for Disease Control and Prevention (KCDC), had previous treatment experience at a participating hospital, and voluntarily consented to participating in this study after receiving a sufficient explanation of the research content, were included. This study is a cohort study, in which the dates of registration and research termination vary among the participants. Data were collected in real time from multiple centers by the Integrative Management System of the KCDC. The optimal date for the repeated investigation was six months after the investigation at the time of cohort registration. However, for participant convenience, a repeated investigation period was added from one to two months after the optimal date. Participants who were not surveyed for over two years after cohort registration were defined as “follow-up loss”. Reasons for ceasing participation, such as death or consented withdrawal, were documented in accordance with the survey format (Figure 2).

Four men were registered by December 2006, and the number of HIV-infected participants consistently increased and reached 1,438 by 2016. Of the 13,584 HIV-infected Koreans in 2016, 13,152 were adults aged 20 years or older. Therefore, about 11% of the HIV-infected patients who were eligible for registration participated in this study (Table 1). Regarding the age distribution, there were 432 HIV-infected Koreans who were less than 20 years of age in 2016, and 11 of these patients participated in this study. About 10% of all infected Koreans between the ages of 20-40 years and 13% of all infected Koreans who were 40 years of age or older participated in this study (Table 2).

Of the 1,438 participants who registered by December 31, 2016, over 50% were diagnosed as HIV positive after 2006. The mean age at cohort registration was 41.5 years, and the mean age at the time of diagnosis was 38.3 years. Of the Korean participants, 1.1% were naturalized foreigners. The immune status at the time of registration was determined by the number of CD4^+^ T cells and HIV RNA. The rates at which these two parameters were measured were 82.1 and 78.6%, respectively. There were 261 patients (18.2%) in the immunodeficiency group, with CD4^+^ T cell numbers less than 200, and 27 patients (1.9%) with HIV RNA numbers of 500,000 or greater (Table 3). According to national reports, only 6,192 Koreans (45.6%) underwent the CD4^+^ T cell test at the time of the report, of whom 2,387 (38.5% of all participants, or 17.6% of all HIV-infected Koreans) were in the immunodeficiency group (CD4< 200) [15].

### Data management

The epidemiology research team manages data in three stages, to assure the data quality. First, the team educates clinical research nurses on standardized guidelines, before data collection. Second, the team performs real-time monitoring and manages the database to minimize errors that can occur during the data collection process. Third, the team statistically reviews the limiting values, outliers, and missing value of data, and develops an algorithm for logical error derivation that can occur from a question or between different investigation timings. Next, the team cleanses the data twice, to estimate errors, and confirms the results with the corresponding hospital. Furthermore, it provides a code book and a guideline on how to use the primitive data, to allow researchers to effectively use the cleansed data, and provides epidemiological consultation or statistical analysis when necessary.

### Research ethics

All participating hospitals give consent for patient participation and provided test result information following IRB approval. Due to the nature of multi-year research projects, the study is continuously reviewed every year. To protect the patients’ personal information, each patient was assigned a cohort management number that did not include personal identification information, such as resident registration number, name, phone number, or address. When receiving data from the KCDC, the purpose of using the data and the date as well as destruction must be clearly stated and fulfilled. In addition, ethics education is regularly held for all researchers.

### Method of investigation

#### Repeated and follow-up investigations

The date of registration varied among the participants, and new participants were recruited every year. For patients for whom the basic investigation was completed, repeated investigations of treatment and disease were conducted every six months. For participants who did not show short-term changes, such as changes in marital statuses and health behaviors, the investigation was repeatedly conducted every 12 months. At least 1,000 participants were maintained every year, and research nurses performed quarterly updates on survey rates. For participants who could not be repeatedly surveyed due to withdrawal or hospital transfer, a follow-up investigation using nationally reported data was used once every year, to investigate whether or not the participants were deceased. By January 2018, a total of 939 participants could be followed-up (excluding those for whom research was terminated).

#### Self-reported questionnaire, and examiner questionnaire

The participants answered a questionnaire that contained questions about basic information, health behaviors (smoking/alcohol use), route of infection, sociopsychological state (depression, anxiety, quality of life), family history, medical history, vaccination history, and symptoms related to HIV/AIDS during the basic investigation period. Medical history, vaccination history, and recent symptoms related to HIV/AIDS were investigated every six months.

Professional research nurses recorded patient treatment histories related to HIV/AIDS and associated opportunistic infections by referring to medical records and patient interviews, instead of using self-reported questionnaire surveys. The date of prescription and types of prescribed medications were recorded in detail. After the basic investigation period, all diseases that occurred after HIV diagnosis were recorded, in chronological order. In the repeated investigation, histories of diseases that occurred after the last investigation were recorded, and data were collected over time (Table 4).

#### Body measurements and clinical examinations

The participants were adults aged 18 years or older, with no significant predicted height changes, and height was assessed in the basic investigation only. However, parameters that were prone to changes such as weight, waist circumference, and hip circumference were measured every six months, in accordance with the guidelines. All body measurements were recorded up to the first decimal place.

It was recommended to record parameters that were examined during chest X-rays, in addition to a radiologist’s comments, and regularly assess these parameters every six months. However, if this was not possible, the assessment must be performed at least once per year. In a diagnostic test that injects purified protein derivatives isolated from a *Mycobacterium tuberculosis* culture and examines the delayed hypersensitivity reaction caused by memory T cells, the diameter of the lower arm, vertical to the major axis, is measured in mm. If the result is positive, the investigation is not repeated; however, investigation is repeated every six months if the result is negative. The cervical pathological examination results were investigated and classified as normal or abnormal. Although the enzyme linked immuno-spot and QuantiFERON tests are not recommended for regular follow-up, they may be prescribed by the physician and performed every six months. Just like the purified protein derivatives of tuberculin skin test, they are not performed again if the result is positive and performed regularly if the result is negative. If a patient showed a response during the qualitative Treponema pallidum test, the quantified value was recorded. The standard unit for the immune test is 1 mm^3^ per number of cells, and the standard unit for viral load is copies/mL. In the patients for whom international units are used, these units must be converted to standard units. If no virus is detected, “0” was recorded. For the complete blood count test, only the test results were recorded using standard units. For hepatitis testing, if the qualitative test result was positive, we recommended to record quantitative test results as well, and the test was not repeated. If the result was negative, the test was performed regularly, every six months, at the discretion of the physician. Some parameters were assessed only during the basic investigation. For general chemical tests, the results of blood collections that were performed in the fasting state were recorded. The estimated glomerular filtration rate was automatically calculated, based on the registered values (gender, age, blood creatinine levels), using the isotope dilution mass spectrometry-modification of diet in renal disease equation (Table 4).

#### Obtaining biological resources

It was recommended to collect biological resources at the time of each investigation. The institution providing the resources was responsible for shipping and producing biological resource (peripheral blood mononuclear cells, plasma) samples. Blood was collected into two cell preparation tubes (8 mL each) then shipped and processed on the same day as the collection. For hospitals in rural areas, under certain circumstances, samples were allowed to be shipped on the same day as the collection then processed the next day, under certain circumstances. For sample preservation, samples were regularly sent to the Korea Biobank of the KCDC and permanently stored (Figure 2).

## MAJOR FINDINGS

A total of 1,483 participants participated in the basic investigation, between December 2006 and February 2018. Nine hundred and eighty-nine participants (66.6%) participated in four or more repeated investigations. The researchers published a total of nine papers in journals, including three on the therapeutic effect of highly active antiretroviral therapy and compliance [18-20], two on sociopsychological factors (anxiety, depression) [21,22], and four on HIV-related diseases, opportunistic infections, hepatitis, and metabolic disorders [23-26]. Several papers on the management of cohort data quality, epidemiological characteristics, route of infection, survival rates of HIV-infected patients and patients with AIDS, simultaneous diagnosis of tuberculosis, and characteristics of proton beam therapy have been written and are being submitted for publication in scholarly journals.

### Advantages/disadvantages

Over 1,000 newly infected patients have emerged on an annual basis since 2013, and the age at infection has been decreasing, with 33.9% of newly infected patients in 2016 found to be in their 20’s [15]. This study is the only HIV/AIDS cohort study that has monitored the incidence of group 3 nationally notifiable communicable infections and the epidemiological and clinical data of patients with HIV/AIDS, requiring management measures collected over time. The data collected over the last 11 years until present (2018) may be used to establish a basis for successful research studies and effective policies for HIV-infected patients in Korea. In 2016, there were 4,004 HIV-infected patients in the 15 participating hospitals, which is equivalent to 29.5% of all Koreans with HIV infection. Thus, by encouraging consistent participation, data representativeness may be secured. In addition, these data can be used to study various topics since not only HIV/AIDS treatment, but also opportunistic infections, drug tolerance, and metabolic disorders were assessed in the early investigation.

However, this study may contain selection bias caused by the lack of active participation by the participants, due to the negative stigma around HIV/AIDS and patients affected by HIV in Korea. The inherent limitations of multi-center cohort studies, and the fact that all participating hospitals were mid- and large-scale hospitals.

## DATA ACCESSIBILITY

Data were distributed only among the researchers who participated in this study, in accordance with the decision of the department of viral diseases of the KCDC, which is the main research institution as of March 2018. Data distribution for external research centers is currently being planned. Data can be used in future research, through a process that complies with cohort data distribution regulations.

## Figures and Tables

**Table 1.** Enrollment by year: December 6, 2006-December 31, 2016

Enrollment year	Korea HIV/AIDS Cohort Study			Korea HIV/AIDS patients^1^		
	Men	Women	Cumulative n (%)	Men	Women	Cumulative n (%)
1985-2005	0	0	0 (0.0)	3,427	355	3,827 (28.2)
2006	4	0	4 (0.3)	687	62	4,576 (33.7)
2007	183	13	200 (13.9)	698	42	5,316 (39.1)
2008	260	19	479 (33.4)	743	54	6,113 (45.0)
2009	142	11	632 (44.1)	710	58	6,881 (50.7)
2010	220	18	870 (60.6)	723	50	7,654 (56.3)
2011	175	12	1,057 (73.5)	827	61	8,542 (62.9)
2012	110	9	1,176 (81.8)	808	60	9,410 (69.3)
2013	58	3	1,237 (86.0)	946	67	10,423 (76.7)
2014	93	1	1,331 (92.6)	1,016	65	11,504 (84.7)
2015	56	8	1,395 (97.0)	974	44	12,522 (92.2)
2016	40	3	1,438 (100.0)	1,002	60	13,584 (100.0)

1 From Cho et al. HIV/AIDS notifications in Korea, 2016 [15].

**Table 2.** Age at enrollment: December 6, 2006-December 31, 2016

Age (yr)	Korea HIV/AIDS Cohort Study	Korea HIV/AIDS patients^1^	Ratio (%)
<20	11 (0.01)	432 (0.03)	3
20-29	267 (0.19)	3,523 (0.26)	8
30-39	389 (0.27)	3,699 (0.27)	11
40-49	389 (0.27)	3,000 (0.22)	13
50-59	251 (0.17)	1,922 (0.14)	13
60+	131 (0.09)	1,008 (0.07)	13
Total	1,438 (100.0)	13,584 (100.0)	11

Values are presented as number (%).

1 From Cho et al. HIV/AIDS notifications in Korea, 2016 [15].

**Table 3.** Baseline characteristics: December 6, 2006-December 31, 2016

Characteristics	Total	Men	Women	p-value
Total	1,438 (100.0)	1,341 (93.3)	97 (6.7)	
Age at enrollment (yr)				
Mean±SD	41.5±12.5	41.2±12.5	45.4±13.1	0.001^1^
Median (IQR)	41 (32-50)	41 (32-50)	48 (34-55)	
Age at diagnosed HIV (yr)	1,432/1,438 (99.6)	1,335/1,341 (99.6)	97/97 (100.0)	
Mean±SD	38.3±12.5	38.0±12.4	41.8±13.9	0.01^1^
Median (IQR)	37 (28-46)	37 (28-46)	41 (30-53)	
Area of origin	1,437/1,438 (99.9)	1,340/1,341 (99.9)	97/97 (100.0)	
Korean	1,423 (98.9)	1,337 (99.7)	86 (88.7)	<0.001^2^
Foreigner	14 (1.0)	3 (0.2)	11 (11.3)	
Year of HIV diagnosis	1,432/1,438 (99.6)	1,335/1,341 (99.6)	97/97 (100.0)	
Prior to 1990	3 (0.2)	3 (0.2)	0 (0.0)	0.90^2^
1990-1999	76 (5.3)	69 (5.2)	7 (7.2)	
2000-2005	392 (27.3)	364 (27.1)	28 (28.9)	
2006-2010	644 (44.8)	601 (44.8)	43 (44.3)	
2011-2012	153 (10.6)	145 (10.8)	8 (8.3)	
2013-2016	164 (11.4)	153 (11.4)	11 (11.3)	
CD4 cell count at enrollment (cell/mm^3^)	1,180/1,438 (82.1)	1,099/1,341 (82.0)	81/97 (83.5)	
Median (IQR)	371 (219-532.5)	370 (218-526.0)	405 (244-594.0)	0.26^1^
≥500	341 (23.7)	310 (23.1)	31 (32.0)	
350-499	289 (20.1)	276 (20.6)	13 (13.4)	0.11
200-349	290 (20.2)	268 (19.9)	22 (22.7)	
<200	260 (18.1)	245 (18.3)	15 (15.5)	
Viral load at enrollment (copies/mL)	1,130/1,438 (78.6)	1,052/1,341 (78.4)	78/97 (80.4)	
Median (IQR)	75 (20-15,867)	75 (20-16,000)	75 (19-14,000)	0.48^1^
>500,000	27 (1.9)	27 (2.0)	0 (0.0)	
1,000-500,000	418 (29.1)	388 (28.9)	30 (30.9)	0.36^2^
400-1,000	46 (3.2)	45 (3.4)	1 (1.0)	
≤400	639 (44.4)	592 (44.1)	47 (48.5)	

Values are presented as number (%).

IQR, inter-quartile range.

1 Wilcoxon rank-sum test.

2 Fisher’s exact test.

**Table 4.** Korea HIV/AIDS Cohort Study questionnaires

Category	Factors
Self-administered questionnaires	
Sociodemographic status	ID, gender, date of birth, race (ethnicity), marital status
Health-related lifestyle	Smoking and drinking habits (smoking status, duration of smoking, drinking status, duration of drinking, etc.)
Transmission route	Sexuality, infection route
Psychosocial status	Beck Depression Inventory, State-Trait Anxiety Inventory, EuroQol 5 dimensions (2015-2018), World Health Organization quality of life-HIV (2018-)
Past disease history	Past disease history (hypertension, diabetes mellitus, lipodystrophy, dyslipidemia, ischemic heart disease, cerebrovascular accident, peripheral vascular disease, hepatitis B, hepatitis C, cancer, TBc, etc.)
Dietary supplement history (-2014)	Multivitamin, vitamin C, vitamin E, beta-carotene, calcium, iron, red ginseng, Chinese medicine, others
Vaccination	Vaccination (BCG, hepatitis B, pneumococcal influenza, tetanus)
Family history	Family history of disease
HIV/AIDS symptoms	Acute HIV related symptoms, current HIV related symptoms
Investigator-administered questionnaires	
	AIDS related disease
	Current & past HARRT history
	ART compliance
	HARRT genotypic resistance (NRTI, NNRTI, major PI mutation)
	Opportunistic infections
	Current & past TBc medical history
	Current & past CMV medical history
Physical and clinical examinations	
Physical examination	Height (cm), weight (kg), waist circumference (cm), hip circumference (cm)
Clinical examination	Blood pressure (mmHg)
	Chest X-ray, PPD skin test, Pap smear, TBc ELISPOT and QuantiFERON, quantitative and qualitative syphilis lymphocyte tests, CD3, CD4, CD8
Viral load	HIV RNA titer
CBC	WBC, Hb, Hct, platelets
Viral hepatitis	HBsAg, Anti-HBs, Anti-HBc, Anti-HCV, Anti-HAV IgG, Anti-Hbe, HBeAg, HBV-DNA, HCV-PCR
CMV	CMV IgG, CMV IgM, CMV RT PCR, CMV Ag
Blood chemistry	FBS, Total-C, LDL-C, HDL-C, TG, AST, ALT, ALP, GGT, T-B, BUN, Cr, eGFR

BCG, bacillus Calmette–Guérin; HIV, human immunodeficiency virus; AIDS, acquired immune deficiency syndrome; HAART, highly active antiretroviral therapy; ART, antiretroviral therapy; NRTI, nucleoside reverse transcriptase inhibitor; NNRTI, non-nucleoside reverse transcriptase inhibitor; PI, protease inhibitor; CMV, cytomegalovirus; PPD, purified protein derivative; TBc, tuberculosis; WBC, white blood cell; Hb, hemoglobin; Hct, hematocrit; HBsAg, hepatitis B antigen; Anti-HBs, anti-hepatitis B surface antibody; Anti-HBc, anti-hepatitis B core antibody; Anti-HCV, anti-hepatitis C virus; Anti-HAV IgG, anti-hepatitis A virus antibody immunoglobulin G; Anti-Hbe, anti-hepatitis B e-antigen; HBeAg, hepatitis B e-antigen; HBV-DNA, hepatitis B virus DNA detection test; HCV-PCR, hepatitis C virus-polymerase chain reaction test; CMV IgG, cytomegalovirus immunoglobulin G test; CMV IgM, cytomegalovirus immunoglobulin M; CMV RT PCR, cytomegalovirus real time polymerase chain reaction test; CMV Ag, cytomegalovirus antigen; FBS, fasting blood sugar; Total-C, total cholesterol; LDL-C, low density lipoprotein cholesterol; HDL-C, high density lipoprotein cholesterol; TG, triglyceride; AST, aspartate aminotransferase; ALT, alanine aminotransferase; ALP, alkaline phosphatase; GGT, gamma(γ) glutamyl transferase; T-B, total bilirubin; BUN, blood urea nitrogen; Cr, creatinine; eGFR, estimated glomerular filtration rate.

## SUPPLEMENTAL MATERIALS

Supplementary Material: Korean version is available at http://www.e-epih.org/.
