# Supplementary material for: Korea HIV/AIDS Cohort Study: study design and baseline characteristics
Source: Epidemiol Health. 2018 Jun 6;40:e2018023. doi: 10.4178/epih.e2018023 (PMC6178365; doi:10.4178/epih.e2018023)
Supplement: Supplementary file 1 [file epih-40-e2018023-supplementary.pdf]

Volume: 40, Article ID: e2018023

https://doi.org/10.4178/epih.e2018023

Korea HIV/AIDS Cohort Study: study design and baseline characteristics

한국 HIV/AIDS 코호트 연구 설계 방법

Bo Youl Choi[1), Jun Yong Choi[2)], Sang Hoon Han[2)], Sang Il Kim[3)], Mee-Kyung Kee[4)], Min Ja Kim[5)], Shin-Woo Kim[6)], Sung Soon Kim[7)], Yu-Mi Kim[8)], Nam Su Ku[2)], Jin-Soo Lee[9)], Joo-Shil Lee[10)], Yunsu Choi[1)], Kyong Sil Park[1),11)], Joon Young Song[12)], Jun Hee Woo[13)], Moon Won Kang[3] , June Kim[2)]

1) Hanyang University School of Medicine, Preventive Medicine, Seoul, Republic of Korea
2) Yonsei University College of Medicine, Department of Internal Medicine and AIDS Research Institute, Seoul, Republic of Korea
3) Division of Infectious Disease, Seoul St. Mary's Hospital, College of Medicine, The Catholic University of Korea, Seoul, Republic of Korea
4) Korea National Institute of Health, Division of Viral Disease Research, Center for Infectious Diseases Research, Chungbuk, Republic of Korea
5) Department of Internal Medicine, Division of Infectious Diseases, Korea University College of Medicine, Seoul, Republic of Korea
6) Kyungpook National University School of Medicine, Internal Medicine, Daegu, Republic of Korea
7) Korea National Institute of Health, Center for Infectious Diseases Research, Chungbuk, Republic of Korea
8) Dong-A University College of Medicine, Preventive Medicine, Busan, Republic of Korea
9) Inha University College of Medicine, Internal Medicine, Incheon, Republic of Korea
10) Center for Immunology and Pathology, Chungbuk, Republic of Korea
11) Hanyang University School of Nursing, Department of Nursing, Seoul, Republic of Korea
12) Korea University College of Medicine, Internal Medicine, Seoul, Republic of Korea
13) University of Ulsan College of Medicine, Infectious Diseases, Seoul, Republic of Korea

Corresponding author: June Kim

Department of Internal Medicine, Yonsei University College of Medicine

50-1 Yonsei-ro, Seodaemun-gu, Seoul, South Korea

Tel: +82 2 2228 1946, Fax: +82 2 393 6884, E-mail: jmkim@yuhs.ac / ORCID: 0000-0002-0337-8573

Volume: 40, Article ID: e2018023

https://doi.org/10.4178/epih.e2018023

초록

1985년 국내 HIV/AIDS 최초 감염인 발생 이후 계속해서 증가하여, 2016년 전체 감염인 수는 15,208명에 이르렀다. 2013년 이후에는 매 해 1,100여명의 신규 감염자가 발생하고 있다. 한국 HIV/AIDS 코호트 연구(Korea HIV/AIDS Cohort Study; KoCosHIV)는 2006년 12월 국내 HIV 감염인의 과학적 근거 기반의 예방, 치료 및 효과적인 관리를 위해 설립되었다. 본 연구는 10년 간 국내 HIV/AIDS 감염인의 약 10%에 해당하는 1,438명의 대상자를 추적 관찰해오면서 1) HIV/AIDS 코호트 기반 연구 구축을 위한 운영체제, 2) 연구방법 및 조사 도구(Case Report Form; CRF) 표준화, 3) 다 기관 코호트 자료로써 수집된 자료의 표준화 및 정제 방법에 대해 개발하였다. 본 연구에서는 종결조사자를 제외한 추적관찰대상자를 연간 1,000여명 이상으로 유지할 것을 목표로 하고 있다(2018년 1월 추적 가능 대상 수; 939명). 2016년 12월까지 남자 93.3%와 여자 6.7%가 포함되어있고(성비 13.9:1), 피험자의 98.9%는 한국인이다. 대상자의 50%이상이 2006년 이후 HIV 양성 확진을 받았다. 이 연구는 HIV 감염인을 위한 만성감염질환의 관리 및 예방 정책을 개발하기 위한 경쟁력 있는 장기과제로 지난 10년 이상에 걸쳐 수집된 자료는 HIV 치료 및 건강증진을 개선하기 위한 지표를 개발하는데 기반이 될 것이다.

Key words: HIV/AIDS, 코호트 연구, 성매개감염병 코호트 연구, 감염병 코호트 연구

Volume: 40, Article ID: e2018023

https://doi.org/10.4178/epih.e2018023

1. 서론

1980-1981 년에 미국 로스앤젤레스를 비롯한 여러 도시의 젊은 동성애 남자에서 주폐포자충 폐렴과 카포시 육종을 일으키는 후천성면역결핍증후군(AIDS; acquired immune deficiency syndrome)이 인지된 뒤 1983 년에 처음으로 이 증후군 환자로부터 병원체를 분리하여 LAV (lymphadenopathy associated virus)라고 명명하였고 [1, 2, 3], 이후 연구자들은 이 면역저하 질환을 HIV (human immunodeficiency virus) 감염에 의한 AIDS 로 명명하였다 [4-6].

1983 년 미국에서 성 정체성이 동성 혹은 양성인 고위험군 남성을 대상으로 HIV 감염과 AIDS 의 발현, 이후 사망에 이르기까지의 질병의 진행 과정을 파악하기 위해 다기관 AIDS 코호트 연구인 MACS(The multicenter AIDS cohort study) 연구를 시작하였고 [7], 이후 1984 년 네덜란드의 암스테르담 코호트 연구(ACS; The Amsterdam cohort study) [8] 와 1988 년 스위스 HIV 코호트 연구(SHCS; The Swiss HIV Cohort study)가 시작되었다 [9]. 발견 초기 구축된 세계 각국의 HIV/AIDS 코호트 연구 결과를 통해 국가와 인종 별로 감염경로, 면역학적 특성, 기회감염 및 사망 원인과 AIDS 발병의 특성이 다르다는 것이 확인되었다 [10-12]. 2003 년 브라질에서는 HIV 브라질 코호트(The HIV-Brazil Cohort)연구를 구축하여 브라질 전역 26 개 공중보건 시설에서의 HIV 감염인을 추적하는 다기관 코호트 연구를 수행 중이다 [13].

최근 UNAIDS (The Joint United Nations Programme on HIV/AIDS)의 자료에 따르면 2016 년 성인 HIV/AIDS 생존 감염인의 수는 약 3,670 만 건이고, 약 180 만 명의 신규 감염인의 발생과 100 만 명의 AIDS 관련 사망이 있었다고 보고하였다 [14].

우리나라는 처음으로 양성 확진이 보고된 1985 년 2 명(내국인 1 명, 외국인 1 명) 이후 2016 년까지 지난 30 여 년간 누적감염자가 15,208 명(내국인 13,584 명, 외국인 1,624 명)으로 증가하였다. 내국인 누적 감염자 13,584 명 중 남자가 12,606 명(92.8%), 여자 978 명(7.2%)으로 남자의 비율이 월등히 높다. 이들 중 2,145 명이 사망하여 누적 사망률은 15.8%로 2016 년 생존 감염인 수는 11,439 명이다. 전 세계적으로 HIV 신규 감염인의 수는 감소하는 추세이지만 2000 년 이후 우리나라 HIV 감염인 수는 약 4 배 증가하였고, 특히 2013 년 이후 매년 1,100 명 이상의 신규 감염인이 발생하였다 [14, 15]. 한편, 국내 HIV/AIDS 연구는 대부분이 특정 병원의 감염자와 환자를 대상으로 진행되었고, 주로 치료 효과에 집중되었다 [16, 17].

이 연구의 목적은 외국과 다른 역학적 특성을 보이는 우리나라의 HIV/AIDS 감염인과 환자를 대상으로 초기 HIV 감염에서 AIDS 발병, 사망에 이르기까지의 자연사를 파악하고, 역학적 임상적 특성을 밝히며, 이에 영향을 미치는 요인을 파악하여 관리와 치료 지침을 개발하는 것이다. 이를 위하여 연구자들은 2006 년부터 HIV/AIDS 감염인을 진료하는 전국의 21 개 의료기관이 참여한

“한국 HIV/AIDS 코호트 연구(Korea HIV/AIDS Cohort Study)”를 시작하였고, 현재까지 1,438 명의 환자 코호트를 구축하여 반복 조사를 시행하고 있다.

2. 연구 내용과 방법

2.1. 참여 병원

한국 HIV/AIDS 코호트 연구는 2006 년부터 2016 년 12 월까지 총 21 개 병원이 참여하였다. 이 중 현재(2018 년) 유지 중인 병원은 전국 6 개 시도에 분포한 15 개 중대형 종합병원이다.

이 연구는 총괄(책임연구원)과 3 개 연구 센터의 연구원, 그리고 역학팀, 질병관리본부 국립보건연구원 바이러스질환연구과로 구성되어 있다. 연구의 전체적인 집행을 위해 총괄에서는 연구 수행, IRB (Institutional Review Board) 승인 획득 및 수집 등 연구와 관련된 행정업무를 담당한다. 역학팀에서는 표준화된 설문조사서와 지침서를 개발하고, 자료 정제 및 역학 컨설팅과 통계지표제공, 통계분석지원 등 자료 활용에 관한 연구 지원을 담당한다. 질병관리본부는 동의서 관리와 자료의 사용 등 권한을 포함하여 연간 연구 진행 상황을 관리하고, 코호트 관리 번호를 지정하며 연구참여자의 기반 및 반복 조사와 생물자원 표본을 관리한다(Fig 1). 코호트의 효과적인 진행을 위해 주기적으로 운영•실무위원진이 모여 연구에 대한 의견을 나누고 지속해서 조사 도구와 지표를 수정•보완한다.

2.2. 연구 대상자

본 연구의 대상자는 HIV 웨스턴 블롯(Western blot) 검사에 의한 양성 확진을 받고 질병관리본부에 등록 후 연구 참여 병원에서 진료 경험이 있는 자로 조사 내용을 충분히 설명 듣고 자발적으로 동의하는 대한민국 국적의 만 18 세 이상 성인 HIV 감염인에 해당한다. 이 연구는 모든 참여자의 등록과 종료 시점이 다른 코호트 연구이며 질병관리본부 통합관리시스템에 의해 다기관에서 조사된 자료를 실시간으로 수집한다. 코호트 등록 시점 기반 조사를 수행한 이후 최적의 반복조사방문일은 6 개월로 정의하고 있으나, 연구 대상자의 편의성을 위해 최적 방문일 기준 1 개월 전부터 2 개월 후까지의 반복 설문 기간을 둔다. 코호트 등록 후 2 년(4 회) 이상 조사에 참여하지 않은 자는 “탈락”으로 정의한다. 이 외의 사망, 동의 철회 등의 연구 참여 중단 사유는 조사서 양식에 따라 작성한다(Fig 2).

2006 년 12 월 연구등록자는 남성 4 명이었으나, 이후 꾸준히 증가하여 2016 년까지 1,438 명의 HIV 감염인이 연구에 참여하였다. 2016 년까지 내국인 신고 감염인 13,584 명 중 20 세 이상 성인이 13,152 명인 점을 빌면, 등록 가능 대상자 중 약 11%에 해당하는 감염인이 연구에

https://doi.org/10.4178/epih.e2018023

참여했음을 알 수 있다(Table 1). 연령군 별로 보았을 때, 2016 년 기준 20 세 미만 내국인 감염자 수는 432 명이고, 그중 11 명이 연구에 참여 중인 것으로 파악되었다. 20~40 세에서는 전체의 약 10%가, 40 세 이상 내국인 감염자 중에서는 13%가 연구에 참여하였다(Table 2).

2016 년 12 월 31 일까지 연구 참여자 1,438 명 중 50% 이상이 2006 년 이후 양성 진단을 받았다. 코호트 등록 시점 평균 연령은 41.5 세였고, 진단 시점 평균 연령은 38.3 세였다. 연구참여자의 98.9%가 한국인(1.1%는 귀화한 외국인)이었다. 등록 시 면역 상태는 CD4 T 세포 수와 HIV RNA 로 판단하며, 각각의 조사율은 82.1%, 78.6%였다. 등록 시점 CD4 T 세포 수가 200 미만인 면역 저하 군은 261 명(18.2%)이고, HIV RNA 가 50 만 이상인 군은 27 명(1.9%)이었다(Table 3). 국가 신고 현황에 따르면 신고 시점 CD4 T 세포 검사를 시행한 내국인 감염인은 6,192 명(45.6%)에 불과했고, 그중 면역 저하 군(CD4<200)은 2,387 명으로 38.5%(전체 내국인 감염인의 17.6%)였다 [15].

2.3. 자료 관리

역학팀은 자료 질 관리를 위해 3 단계로 자료 관리를 수행한다. 첫째, 자료 수집 전 단계에서 연구 간호사를 위한 표준화 지침 교육을 하고, 둘째, 자료 수집 단계에서 발생할 수 있는 오류를 최소화하기 위하여 실시간 모니터링과 데이터베이스를 관리한다. 셋째, 수집된 자료의 극한값, 이상치, 누락 여부를 통계적으로 검토하고, 문항 혹은 회차 사이에서 발생할 수 있는 논리적 오류 도출을 위한 알고리즘을 개발한다. 이후 연 2 회 자료 정제를 시행하여 오류 예상값을 도출하고 해당 병원에 확인을 요청한다. 더불어 연구자가 정제된 자료를 효과적으로 활용할 수 있도록 코드북 및 원시 자료 이용지침서를 제공하고, 때에 따라 역학 자문이나 통계 분석을 지원한다.

3. 연구 윤리

모든 참여 연구 병원은 IRB 승인 후 환자의 연구 참여 및 검사에 대한 정보 제공 동의를 받는다. 다년도 연구과제의 특성상 매년 연구내용에 관하여 연구 지속심의를 받는다. 환자의 개인정보보호를 위해 개별 코호트 관리 번호를 부여하고, 주민등록번호, 실명, 전화번호 혹은 주소와 같은 개인식별정보는 포함하지 않는다. 질병관리본부로부터 자료를 내려받을 때 활용 목적과 파기 기한을 명시하고 지킨다. 또한, 윤리 교육은 정기적으로 전체 참여 연구원을 대상으로 시행한다.

4. 조사 내용과 방법

4.1. 반복 조사와 추적 조사

Volume: 40, Article ID: e2018023

https://doi.org/10.4178/epih.e2018023

모든 연구대상자의 등록 시점이 다르며, 매년 새로운 대상자를 추가로 모집하고 있다. 기반 조사 시 모든 문항에 대해 조사한 후 치료 및 질병 관련 문항의 경우 6 개월 주기로 반복 조사하고, 결혼상태 및 건강행태 등과 같이 단기간 내 변화가 없는 문항의 경우 12 개월 주기로 반복 조사한다. 연간 1,000 명 이상의 참여자를 유지하고, 탈락을 최소화하기 위하여 분기별로 연구간호사에게 조사율을 공지하여 관리 중이다. 탈락 혹은 전원 등의 사유로 반복 조사가 불가능한 자에 대해 국가 신고 자료를 활용하여 사망 여부를 확인하는 추적 조사를 연 1 회 실시한다. 2018 년 1 월까지 총 939 명의 추적 가능한 연구 대상자(연구 종결된 참여자 제외)가 있다.

4.2. 자가 작성 설문 및 조사자 설문

기반 조사 시점에 기본정보, 건강행태(흡연/음주력), 감염경로, 사회심리상태(우울, 불안, 삶의 질), 가족력, 과거질환력과 예방접종력 그리고 HIV/AIDS 관련 증상을 포함한 설문조사에 직접 응답한다. 과거질환력, 예방접종력과 최근 HIV/AIDS 관련 증상은 6 개월 주기로 반복 조사한다.

자가 작성 설문조사와 달리 HIV/AIDS 와 관련된 자세한 치료력 및 관련 기회감염 등에 대하여 의무 기록과 환자 면담을 참고하여 전문 연구간호사가 직접 입력한다. 해당 부분은 의무 기록에 기반을 두어 처방 일자와 약물 제제의 종류 등을 자세히 기록하게 되어있다. 기반 조사 시점에는 HIV 진단 이후 모든 병력에 대해 시간순으로 기록하고, 반복조사에서는 지난 조사 이후 병력에 대해 기록하여 시간의 흐름에 따른 자료를 구축하였다(표 4).

4.3. 신체 계측 및 임상검사

연구 참여자는 만 18 세 이상의 성인으로 신장에 큰 변함이 없을 것으로 예상하고 기반 조사 시에만 조사한다. 하지만 체중, 허리둘레, 엉덩이둘레 등 변화가 가능한 조사 항목은 지침에 따라 6 개월마다 측정한다. 신체 계측 값은 모두 소수점 첫째 자리까지 측정하는 것을 원칙으로 한다.

흉부 방사선 검사는 방사선의학과의 소견과 함께 실제 진단된 해당 항목을 함께 입력하고, 6개월 주기 조사를 권장하지만 부득이한 경우 적어도 연 1회 검사한다. 결핵균 배양액으로부터 분리한 PPD (Purified protein derivative)를 피 내 주사하여 기억 T림프구에 의한 지연 과민반응을 확인하는 결핵 피부 반응 진단 검사는 아래팔의 장축에 수직이 되는 지름 길이를 측정하고 mm 기준으로 조사한다. 양성이면 반복 조사를 시행하지 않으며, 음성이면 매 6개월 주기로 검사한다. 자궁경부 병리 검사는 도출된 소견을 포함하여 검사결과를 정상 혹은 이상으로 구분하여 조사한다. 결핵균 감염에 대한 ELISPOT (Enzyme Linked Immuno-spot) 및 Quantiferon 검사는 정규 추적 검사 권장 항목은 아니나, 주치의의 처방에 따르며 매 6개월 주기로 입력할 수 있다. 결핵 피부 반응 검사와 마찬가지로 양성이면 이후 반복 조사를 시행하지 않으며, 음성이면 이후 반복 조사를 시행한다. 매독균 감염의 정성검사에서 반응이 있으면, 정량 값을 입력한다. 면역 검사의 기준 단위는 1mm^3당 세포 수(cells)로 표기한다.

바이러스양의 경우 코호트 자료 내 기준 단위는 copies/mL를 기준으로 하나, 각 병원 병리과 보고에 따라 IU (International Units)를 사용하는 경우 환산 후 기재하고, 검출되지 않으면 “0”으로 기재한다. CBC 검사는 기준 단위에 맞게 검사결과만을 입력한다. 간염 검사의 경우 정성 검사가 양성이면 정량검사결과 값까지 기재하는 것을 권장한다. 이 경우 반복 조사를 시행하지 않으며, 음성이면 주치의의 판단에 따라 6개월 주기로 반복 조사를 시행한다. 일부 문항의 경우 기반 조사 시에만 검사한다. 일반화학검사는 반드시 공복 상태에서 시행한 채혈 결과를 기재하고, 사구체 여과율(eGFR)의 경우 IDMS-MDRD (isotope dilution mass spectrometry-modification of diet in renal disease) 계산식에 따라 기존 입력 결과(성별, 나이, 혈청 크레아티닌 결과)를 통해 자동 계산된다(표 4).

4.5. 생물 자원 확보

조사 시점마다 생물 자원의 수집을 권장하며, 위탁기관에서는 운송 및 생물자원의 시료 제작(PBMC, Plasma)을 일괄하여 담당한다. 채혈 시 CPT (cell preparation tube) 2 개(각 8mL)를 수집하고, 당일 운송 및 처리를 원칙으로 한다. 때에 따라 지방에 있는 병원은 당일 운송 후 다음 날 생물 자원 처리를 진행할 수 있다. 시료보존을 위해 정기적으로 질병관리본부 인체 자원은행에 보내 영구보관한다(그림 2).

5. 주요 연구 성과

2006 년 12 월부터 2018 년 2 월까지 총 1,483 명의 연구참여자가 기반 조사를 시행하였고, 4 회 이상 반복 조사를 시행한 연구참여자는 988 명(66.6%)이다. 지금까지 연구자들은 총 9 편의 논문을 학술지에 게재하였는데, HAART 치료 효과와 내과 및 순응도 관련 3 편 [18-20], 사회심리학적 요인(우울, 불안) 관련 2 편 [21, 22], HIV 관련 질환 및 기회감염, 간염, 대사 이상 관련 4 편 [23-26]이 있다. 현재 코호트 자료 질 관리 및 역학적 특성과 감염경로, HIV 감염인과 AIDS 환자의 생존율, 결핵 동시 감염 진단, 초 치료 특성을 포함한 다양한 연구 논문들을 작성하여 학술잡지에 투고 중이다.

6. 장•단점

2013 년 이후 매년 1,000 여 명 이상의 신규 감염인이 발생하고, 2016 년 신규 감염인의 33.9%가 20 대로 감염 시점 나이는 점차 감소하는 추세이다 [15]. 본 연구는 3 군 법정 감염병으로서 계속해서 발생을 감시하고 관리 대책 마련이 필요한 HIV/AIDS 감염인의 역학 및 임상적 자료를 시간의 흐름에 따라 추적한 결과를 볼 수 있는 국내 유일의 HIV/AIDS 코호트 자료이다. 2018 년 현재까지 만 11 년간 수집된 자료는 앞으로 다양한 관련 연구 성과를 도출하는

https://doi.org/10.4178/epih.e2018023

기반이 되고, 국내 감염인을 위한 효과적인 정책 마련에 도움이 될 것으로 기대한다. 2016 년 당시 15 개 연구 참여 병원 내 HIV 감염인은 4,004 명으로 내국인 신고 감염인 중 29.5%에 해당하는 연구참여자가 포함되어 있어 지속적인 연구 참여 독려를 통해 대표성 있는 자료로 활용할 수 있다. 또한, 초기 구축 시 HIV/AIDS 치료뿐 만 아니라 기회감염, 약제 내성, 대사 질환 요인을 고려한 조사를 시행하였기에 다양한 주제에 관한 연구가 가능하다.

다만, 우리나라 정서상 질병과 감염인에 대한 편견이 강해 연구 참여에 적극적이지 않고, 다기관 코호트 연구가 갖는 한계가 있으며 현재 연구에 참여하는 기관이 모두 중대형 종합병원이라는 점에서 연구 대상자 선정에 편향이 있을 수 있다.

7. 자료 공개

이 연구는 2018 년 3 월 현재 주관 연구기관인 질병관리본부 바이러스질환연구과와 한국 HIV/AIDS 코호트 연구 운영위원회의 결정에 따라 참여 연구원만 자료 분양이 가능하다. 외부 연구원에 대한 자료 분양을 계획 중에 있으며, 추후 코호트 자료 분양 규정에 따른 절차를 통해 연구에 활용할 수 있다.

8. 연구지원

이 연구는 질병관리본부 만성질환코호트사업(한국 HIV/AIDS 코호트 연구)으로 지원받아 수행되었습니다(2016-E51003-02).

9. 한국 HIV/AIDS 코호트 연구 참여기관

참여기관: 아주대학교병원, 서울아산병원, 충북대학교 병원, 이화여자대학교 목동병원, 가천길병원, 한림대학교 강동성심병원, 한림대학교 강남성심병원, 한림대학교 평촌성심병원, 한양대학교 서울병원, 인하대학교병원, 강북삼성병원, 고려대학교 안암병원, 고려대학교 안산병원, 고려대학교 구로병원, 경북대학교 병원, 순천향대학교 서울병원, 가톨릭대학교 서울성모병원, 가톨릭대학교 성빈센트병원, 영남대학교병원, 연세대학교 신촌세브란스병원, 연세대학교 원주세브란스기독병원

데이터센터: 한양대학교 의과대학 예방의학교실

REFERENCES

1. Nelson KE, Celentano DD. Human Immunodeficiency virus infection and the acquired immune deficiency syndrome. In: Nelson KE, Williams CM. Infectious disease epidemiology. 3rd ed. Jones & Barlett Learning; 2014, p. 651-722

2.Montagnier L. Lymphadenopathy-associated virus: From molecular biology to pathogenicity. Annals of Internal Medicine 1985;103:689-693.

3.Barre-Sinoussi F, Chermann J, Rey F, Nugeyre M, Chamaret S, Gruest J, et al. Isolation of a T-lymphotropic retrovirus from a patient at risk for acquired immune deficiency syndrome (AIDS). Science 1983;220:868-871.

4.Friedman A, Freeman W, Orellana J, Kraushar M, Starr M, Luntz M. Cytomegalovirus retinitis and immunodeficiency in homosexual males. The Lancet 1982;319:958.

5.Self PC, Filardo TW, Lancaster FW. Acquired immunodeficiency syndrome (AIDS) and the epidemic growth of its literature. Scientometrics 1989;17:49-60.

6.Control CfD, Prevention. Pneumocystis pneumonia--Los Angeles. 1981. MMWR. Morbidity and mortality weekly report 1996;45:729.

7.Detels R, Jacobson L, Margolick J, Martinez-Maza O, Munoz A, Phair J, et al. The multicenter AIDS cohort study, 1983 to…. Public health 2012;126:196-198.

8.Coutinho RA. The Amsterdam Cohort Studies on HIV infection and AIDS. Journal of acquired immune deficiency syndromes and human retrovirology: official publication of the International Retrovirology Association 1998;17:S4-8.

9.Study SHC, Schoeni-Affolter F, Ledergerber B, Rickenbach M, Rudin C, Günthard HF, et al. Cohort profile: the Swiss HIV Cohort study. International journal of epidemiology 2009;39:1179-1189.

10.Ledergerber B, Egger M, Opravil M, Telenti A, Hirschel B, Battegay M, et al. Clinical

progression and virological failure on highly active antiretroviral therapy in HIV-1 patients: a prospective cohort study. The Lancet 1999;353:863-868.

11.Lichtenstein B, Laska MK, Clair JM. Chronic sorrow in the HIV-positive patient: Issues of race, gender, and social support. AIDS Patient Care and STDs 2002;16:27-38.

12.Egger M, May M, Chêne G, Phillips AN, Ledergerber B, Dabis F, et al. Prognosis of HIV-1-infected patients starting highly active antiretroviral therapy: a collaborative analysis of prospective studies. The Lancet 2002;360:119-129.

13.Grangeiro A, Escuder MM, Cassanote AJF, Souza RA, Kalichman AO, Veloso V, et al. The HIV-Brazil cohort study: design, methods and participant characteristics. PLoS One 2014;9:e95673.

14. Joint United Nations Programme on HIV/AIDS. UNAIDS data 2017 [cited 2018 Jul 13]. Available from: http://www.unaids.org/sites/default/files/media_asset/20170720_Data_book_2017_en.pdf.

15. Choi JH, Kim S, Park P, Cho KS. HIV/AIDS notifications in Korea, 2016 [cited 2018 Jul 31]. Available from: http://www.cdc.go.kr/CDC/cms/content/mobile/90/75790_view.html (Korean).

16.Kim JM, Cho GJ, Hong SK, Chang KH, Chung JS, Choi YH, et al. Epidemiology and clinical features of HIV infection/AIDS in Korea. Yonsei medical journal 2003;44:363-370.

17.Sung H, Jung YS, Kang MW, Bae IG, Chang H-H, Woo JH, et al. High frequency of drug resistance mutations in human immunodeficiency virus type 1-infected Korean patients treated with HAART. AIDS research and human retroviruses 2007;23:1223-1229.

18.Song JY, Lee JS, Jung HW, Choi HJ, Lee JS, Lee J, et al. Primary anti-retroviral resistance in treatment-naive HIV-infected patients: a Korean HIV/AIDS cohort study. Infection and Chemotherapy 2009;41:230-232.

19.Kim MJ, Chang H-H, Kim SI, Kim YJ, Park DW, Kang C, et al. Trend of CD4+ Cell Counts

at Diagnosis and Initiation of Highly Active Antiretroviral Therapy (HAART): Korea HIV/AIDS Cohort Study, 1992-2015. Infection & chemotherapy 2017;49:101-108.

20.Kim MJ, Lee SA, Chang H-H, Kim MJ, Woo JH, Kim SI, et al. Causes of HIV Drug Non-Adherence in Korea: Korea HIV/AIDS Cohort Study, 2006-2015. Infection & chemotherapy 2017;49:213-218.

21.Kee MK, Lee SY, Kim NY, Lee JS, Kim JM, Choi JY, et al. Anxiety and depressive symptoms among patients infected with human immunodeficiency virus in South Korea. AIDS care 2015;27:1174-1182.

22. Park KS. Impact of depression and anxiety on cardiovascular risk using Framingham score among HIV positivie patients: an analysis of the Korea Cohort HIV/AIDS Study. [dissertation]. Seoul: Hanyang University; 2015 (Korean).

23.Kim YJ, Woo JH, Kim MJ, Park DW, Song J-Y, Kim SW, et al. Opportunistic diseases among HIV-infected patients: a multicenter-nationwide Korean HIV/AIDS cohort study, 2006 to 2013. The Korean journal of internal medicine 2016;31:953.

24.Oh DH, Ahn JY, Kim SI, Kim MJ, Woo JH, Kim WJ, et al. Metabolic Complications among Korean Patients with HIV Infection: The Korea HIV/AIDS Cohort Study. Journal of Korean medical science 2017;32:1268-1274.

25.Kim YC, Ahn JY, Kim JM, Kim YJ, Park DW, Yoon YK, et al. Human Immunodeficiency Virus (HIV) and Hepatitis Virus Coinfection among HIV-Infected Korean Patients: The Korea HIV/AIDS Cohort Study. Infection & chemotherapy 2017;49:268-274.

26.Kim EJ, Ahn JY, Kim YJ, Wie SH, Park DW, Song JY, et al. The Prevalence and Risk Factors of Renal Insufficiency among Korean HIV-Infected Patients: The Korea HIV/AIDS Cohort Study. Infection & chemotherapy 2017;49:194-205.

Table 1. Research enrollment by year: December 6, 2006 – December 31, 2016

Year of Research Enrollment	Male	Female	Cumulative N (%)
**2006**	4	0	4(0.28)
**2007**	183	13	200(13.9)
**2008**	260	19	479(33.4)
**2009**	142	11	632(44.1)
**2010**	220	18	870(60.6)
**2011**	175	12	1,057(73.5)
**2012**	110	9	1,176(81.8)
**2013**	58	3	1,237(86.0)
**2014**	93	1	1,331(92.6)
**2015**	56	8	1,395(97.0)
**2016**	40	3	1,438(100.0)

Volume: 40, Article ID: e2018023

https://doi.org/10.4178/epih.e2018023

Table 2. Baseline characteristics: December 6, 2006 – December 31, 2016

	N(%)			*p* value
	Total	Male	Female	
**N**	1,438(100.0)	1,341(93.3)	97(6.7)	
**Age at enrollment(years)**				
Mean ± SD	41.5 ± 12.5	41.2 ± 12.5	45.4 ± 13.1	0.001 [1]
Median (Inter-quartile range)	41(32 - 50)	41(32 - 50)	48(34 - 55)	
**Age at diagnosed HIV(years)**	1,432/1,438 (99.6)	1,335/1,341 (99.6)	97/97 (100.0)	
Mean ± SD	38.3 ± 12.5	38.0 ± 12.4	41.8 ± 13.9	0.011 [1]
Median (Inter-quartile range)	37 (28 - 46)	37 (28 - 46)	41 (30 - 53)	
**Area of Origin**	1,437/1,438(99.9)	1,340/1,341(99.9)	97/97(100.0)	
Korean	1,423(98.9)	1,337(99.7)	86(88.7)	<0.0001 [2]
Foreigner	14(1.0)	3(0.2)	11(11.3)	
**Year of HIV diagnosis**	1,432/1,438(99.6)	1,335/1,341(99.6)	97/97(100.0)	
Prior to 1990	3(0.2)	3(0.2)	0(0.0)	
1990-1999	76(5.3)	69(5.2)	7(7.2)	
2000-2005	392(27.3)	364(27.1)	28(28.9)	0.901 [2]
2006-2010	644(44.8)	601(44.8)	43(44.3)	
2011-2012	153(10.6)	145(10.8)	8(8.3)	
2013-2016	164(11.4)	153(11.4)	11(11.3)	
**CD4 cell count at enrollment (cell/mm3)**	1,180/1,438(82.1)	1,099/1,341 (82.0)	81/97 (83.5)	
Median(IQR)	371 (219 - 532.5)	370 (218 - 526)	405 (244 - 594)	0.262 [1]
≥500	341(23.7)	310(23.1)	31(32.0)	
350-499	289(20.1)	276(20.6)	13(13.4)	0.110
200-349	290(20.2)	268(19.9)	22(22.7)	
<200	260(18.1)	245(18.3)	15(15.5)	
**Viral load at enrollment (copies/mL)**	1,130/1,438 (78.6)	1,052/1,341 (78.4)	78/97 (80.4)	
Median(IQR)	75 (20 - 15,867)	75 (20 - 16,000)	75 (19 - 14,000)	0.483 [1]
>500,000	27(1.9)	27(2.0)	0(0.0)	
1,000-500,000	418(29.1)	388(28.9)	30(30.9)	0.362 [2]
400-1,000	46(3.2)	45(3.4)	1(1.0)	
≤ 400	639(44.4)	592(44.1)	47(48.5)	

[1] Wilcoxon rank-sum test

[2] Fisher's exact test

Table 3. KoCosHIV study questionnaires

Category	Factor
**Self-administered questionnaires**	
**Sociodemographic status**	ID, Gender, Date of birth, Race (Ethnicity), Marital status
**Health-related lifestyle**	Smoking & drinking habits (Smoking status, Duration of smoking, Drinking status, Duration of drinking, etc.)
**Transmission route**	Sexuality, Infection route
**Psychosocial status**	BDI (Beck Depression Inventory), STAI (State-Trait Anxiety Inventory), EQ5D(EuroQol 5 dimensions, 2015~2018), WHOQOL-HIV (world health organization quality of life-HIV, 2018~)
**Past disease history**	Past disease history (Hypertension, Diabetes mellitus, Lipodystrophy, Dyslipidemia, Ischemic heart disease, Cerebrovascular accident, Peripheral vascular disease, Hepatitis B, Hepatitis C, Cancer, Tuberculosis, etc.)
**Dietary supplement history (~2014)**	Multivitamin, Vitamin C, Vitamin E, Beta carotene, Calcium, Iron, Red Ginseng, Chinese medicine, Others
**Vaccination**	Vaccination (BCG, Hepatitis B, Pneumococcal Influenza, Tetanus)
**Family history**	Family history of disease
**HIV/AIDS symptoms**	Acute HIV related symptoms, Current HIV related symptoms
**Investigator-administered questionnaires**	
	AIDS related disease
	Current & Past HARRT history
	ART Compliance
	HARRT genotypic resistance (NRTI, NNRTI, major PI mutation)
	Opportunistic infections
	Current & Past Tuberculosis medical history
	Current & Past CMV medical history
**Physical and clinical examinations**	
**Physical examination**	Height (cm), Weight (kg), Waist circumference (cm), Hip circumference (cm)
**Clinical examination**	Blood pressure (mmHg)
	Chest X-ray, PPD skin test, Pap Smear, TBc ELISPOT and QuantiFERON, Quantitative and qualitative syphilis lymphocyte tests, CD3, CD4, CD8
**Viral load**	HIV RNA titer
**CBC**	WBC, Hb, Hct., platelets
**Viral hepatitis**	HBsAg, Anti-HBs, Anti-HBc, Anti-HCV, Anti-HAV IgG*, Anti-Hbe, HBeAg, HBV-DNA, HCV-PCR
**CMV**	CMV IgG, CMV IgM, CMV RT PCR, CMV Ag
**Blood chemistry**	FBS, Total-C, LDL-C, HDL-C, TG, AST, ALT, ALP, GGT, T-B, BUN, Cr, eGFR

HAART, highly active antiretroviral therapy; ART, antiretroviral therapy; NRTI, nucleoside reverse transcriptase inhibitor; NNRTI, non-nucleoside reverse transcriptase inhibitor; PI, protease inhibitor; CMV, cytomegalovirus; PPD, purified protein derivative; TBc, tuberculosis; WBC, white blood cell; Hb, hemoglobin; Hct, hematocrit; HBsAg, Hepatitis B antigen; Anti-HBs, anti-Hepatitis B surface antibody; Anti-HBc, anti-Hepatitis B core antibody; Anti-HCV, anti-Hepatitis C virus; Anti-HAV IgG, anti-Hepatitis A virus antibody immunoglobulin G; Anti-Hbe, anti-Hepatitis B e-antigen; HBeAg, Hepatitis B e-antigen; HBV-DNA, Hepatitis B virus DNA detection test; HCV-PCR,

Volume: 40, Article ID: e2018023

https://doi.org/10.4178/epih.e2018023

Hepatitis C virus-polymerase chain reaction test; CMV IgG, cytomegalovirus immunoglobulin G test; CMV IgM, cytomegalovirus immunoglobulin M; CMV RT PCR, cytomegalovirus real time polymerase chain reaction test; CMV Ag, cytomegalovirus antigen; FBS, fasting blood sugar; Total-C, total cholesterol; LDL-C, low density lipoprotein cholesterol; HDL-C, High density lipoprotein cholesterol; TG, triglyceride; AST, aspartate aminotransferase; ALT, alanine aminotransferase; ALP, alkaline phosphatase; GGT, gamma(γ) glutamyl transferase ; T-B, total bilirubin ; BUN, blood urea nitrogen ; Cr, creatinine ; eGFR, estimated glomerular filtration rate ;

Volume: 40, Article ID: e2018023

https://doi.org/10.4178/epih.e2018023

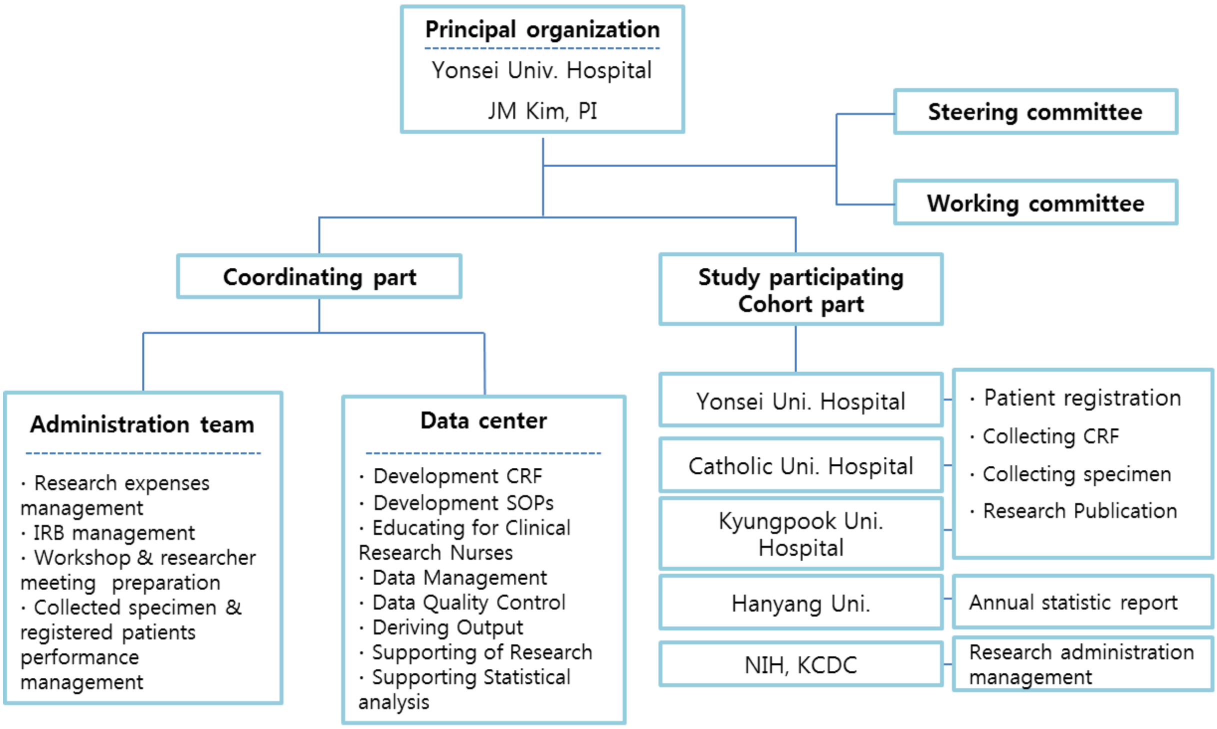

Fig 1. Schematic flow chart of KoCosHIV organization. PI, principal investigator; CRF, case report form; SOP, standard operating procedures.

Volume: 40, Article ID: e2018023

https://doi.org/10.4178/epih.e2018023

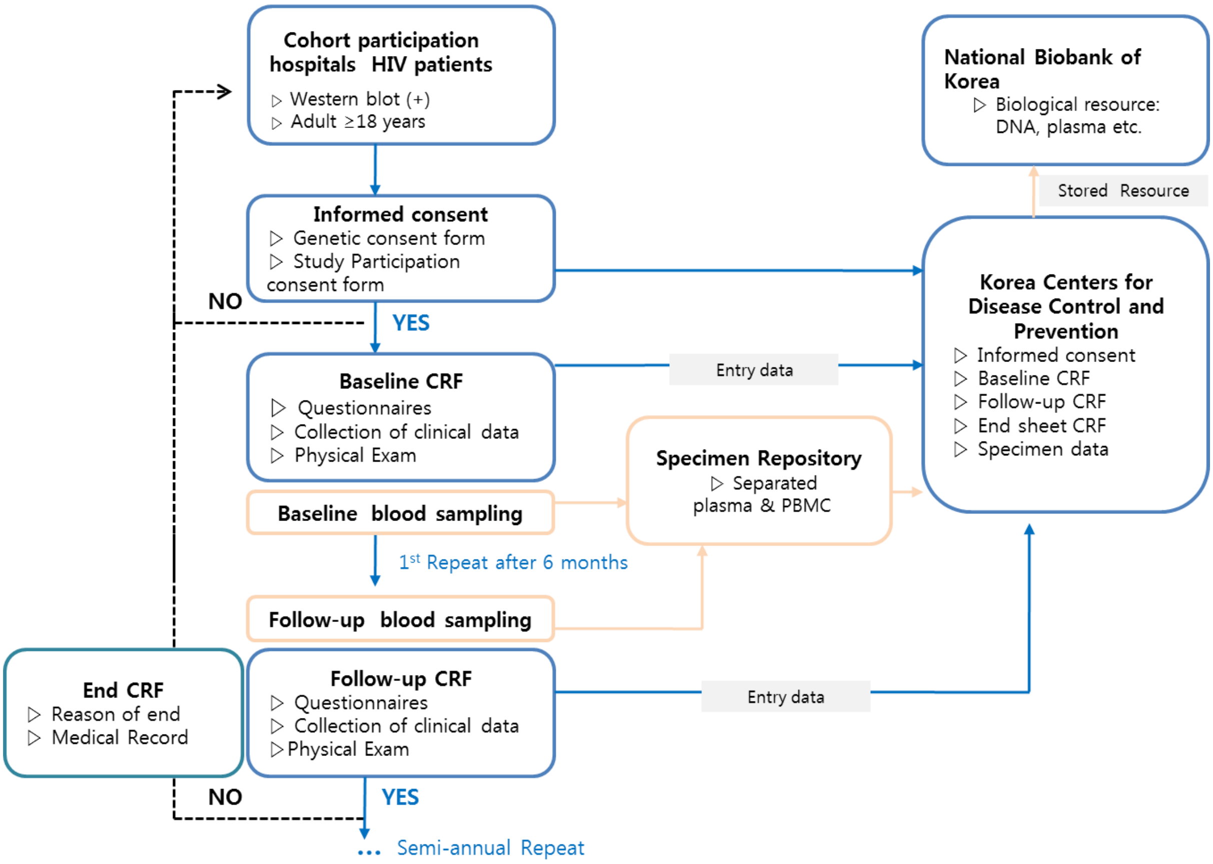

Fig 2. KoCosHIV study design. CRF, case report form; DNA, deoxyribonucleic acid; PBMC, peripheral blood mononuclear cell.
